# Perispinal etanercept: Potential as an Alzheimer therapeutic

**DOI:** 10.1186/1742-2094-5-3

**Published:** 2008-01-10

**Authors:** W Sue T Griffin

**Affiliations:** 1 Geriatric Research, Education and Clinical Center, Neurobiology, Physiology, and Psychiatry, University of Arkansas for Medical Sciences, and the Geriatrics, Education Clinical Center, Central Arkansas Veterans Healthcare System, Little Rock, Arkansas 72205, USA

## Abstract

Tumor necrosis factor-alpha (TNF) is one of a number of systemic and immunomodulating cytokines that generally act to promote acute-phase reactions but can drive degenerative changes when chronically elevated. Traditional focus on TNF has been directed at these inflammation-related functions. Of particular relevance to intersections between neuroinflammation and neurodegeneration is the ability of TNF to increase expression of interleukin-1 (IL-1), which in turn increases production of the precursors necessary for formation of amyloid plaques, neurofibrillary tangles, and Lewy bodies. More recent data have revealed that TNF, one of the few gliotransmitters, has strikingly acute effects on synaptic physiology. These complex influences on neural health suggest that manipulation of this cytokine might have important impacts on diseases characterized by glial activation, cytokine-mediated neuroinflammation, and synaptic dysfunction. Toward such manipulation in Alzheimer's disease, a six-month study was conducted with 15 probable-Alzheimer patients who were treated weekly with perispinal injection of Etanercept, an FDA-approved TNF inhibitor that is now widely used for treatment of rheumatoid arthritis and other systemic diseases associated with inflammation. The results demonstrated that perispinal administration of etanercept could provide sustained improvement in cognitive function for Alzheimer patients. Additionally, the authors were impressed by the striking rapidity with which these improvements occurred in the study patients. An example of this rapid improvement is presented in this issue as a case report by Tobinick and Gross. Such rapid gain of function inspires speculation about the role of gliotransmission or other equally rapid synaptic events in the relationship of TNF to Alzheimer-impacted neurophysiology. Because of the inability of large molecules such as etanercept to cross the blood brain barrier following conventional systemic administration, it is likely that the more direct drug delivery system pioneered by Tobinick also contributed to the effectiveness of the treatment. If so, this system could be useful in drug delivery to the brain in other neural disorders, as well as in animal research studies, many of which currently employ delivery strategies that inflict damage to neural cells and thus engender neuroinflammatory responses.

## Introduction

The Tobinick and Gross case report in this issue of the Journal of Neuroinflammation [1] is hopefully the first of many articles attesting to the benefit of direct-to-the-brain delivery of anti-cytokine therapies, which may result in rapid and sustained improvement in cognition, behavior, and attentiveness. In view of the discouraging results to date of trials testing the efficacy of anti-inflammatory treatments and vaccines directed against A-beta, together with the mounting numbers of new Alzheimer cases each year, the results shown in the case report in this issue and those from previous reports by Tobinick and colleagues [2-4] are indeed welcome. On the clinical research side, these findings call for clinical trials to more completely characterize the efficacy of etanercept treatment and the appropriateness of the delivery system; and on the basic research side, they underscore the need for a clearer picture of the functions of neural cytokines, in particular studies to pinpoint the basic mechanisms underlying not only this rapid recovery of functions but also the underlying principles responsible for maintenance of these functions over months, even years, which these authors report for perispinal treatment with etanercept.

## Background

The rapidity with which cognitive and behavioral functions are recovered by the patient described in a case report in this issue by Tobinick and Gross is consistent with the idea that perispinal etanercept treatment modulates synaptic function. It is tempting to suppose that this rapid recovery of function is a consequence of etanercept capture of excess glia-derived TNF, which results in reversal of synaptic dysregulation. TNF has been described in experimental studies as a gliotransmitter involved in modulation of synapses [5-7] and of long term potentiation and memory functions [8,9]. Interestingly, TNF knockout mice show increased performance in the Morris water-maze test of spatial memory [10,11], and overexpression of TNF is associated with impairment in the same task [11]. Thus, detrimental consequences are to be expected in the case of TNF elevation under conditions of neuroinflammation, of which Alzheimer's disease is but one example. Moreover, titration of these TNF levels back to the normal range by a tool such as perispinal etanercept is a logical approach for effecting cognitive recovery. By analogy, other proinflammatory cytokines that have been shown to interfere with LTP and related behaviors, such as IL-1 [12,13], might be amenable to similarly beneficial manipulations.

It is also worth noting that while this case report emphasizes the rapidity of the response to etanercept, its effects are also relatively long-lasting [3], suggesting involvement of mechanisms that are more classically anti-inflammatory. It is even possible that they involve activity beyond the TNF-binding activity of etanercept. The drug is actually a fusion protein comprising domains from a TNF receptor fused to the Fc portion of IgG_1_. Passive immunization is one of the intriguing developments in prospective AD treatments; and while the preclinical studies and current Phase III trials have focused on delivery of antibodies that are specifically reactive with amyloid beta-peptide (Abeta), it appears that *any *immunization that generates a strong humoral response can facilitate the clearance of Abeta plaques in APP-transgenic mice [14]. Anti-inflammatory effects of certain Fc receptor ligands are well established [15], suggesting that etanercept treatment may have a generalized effect on the immune system due to Fc receptor ligation. Calculations, based on stoichiometry of binding and clearance, suggest the possibility that the effects of anti-Abeta antibodies result from such an antigen-independent mechanism [16]. Exploration of the functional consequences of the Fc fusion domain in etanercept might reveal this agent to be a combination anti-TNF/"passive immunization" therapy.

## Commentator observations

In this section, my comments reflect my own thoughts on this treatment strategy and my own experiences in viewing it, and are accompanied by a few of my own remarks directed toward the necessity of giving attention to this novel treatment. I first became aware of this treatment when a reporter from *The Los Angeles Times *contacted me in 2006 for a comment on an article he was writing on a novel and successful Alzheimer treatment trial. Although my interest was piqued by this, it was only more recently when Dr. Tobinick contacted me and sent a preprint of the findings discussed in the *Times *[3] that I decided to go and see for myself the treatment and talk to the patients and family members. I called the day before, and was invited to visit the next day, November 7, 2007. Each of the three patients I saw treated had been tested and diagnosed with probable Alzheimer's disease by a neurologist before perispinal etanercept treatment had begun. They and their families invited me to be present during the treatment and in the interviews before and after. I noticed clinical improvement in each of the three patients within minutes following treatment. My first impression was that there was a clear, easily discernible, difference in each. They were more cheerful, more at ease, and more attentive. My impressions were the same as those shared by each of the families (please see the movie for example). This rapid turn around brought to mind the first time, now almost two decades ago[17], that I was the original witness to the remarkable overexpression of immune cytokines in activated glia in Alzheimer patients and even in fetuses and neonates with Down's syndrome – I was amazed!

## Conclusion

The most important outcome of this case, in the mind of the commentator, is that we endeavor to reduce the bias that is inherent and important in scientific thinking, but which, unfortunately, can impede timely consideration of new scientific approaches. Recognition of the role of cytokines in the pathogenesis of Alzheimer's disease provided a new paradigm for the Alzheimer research community [18]. And now, the therapeutic intervention in this case report provides a new approach to regulation of the role that cytokines play in pathogenesis. In view of this, constructive scientific approaches to examine and further investigate the beneficial results reported for perispinal etanercept treatment should be undertaken. Taking advantage of what we have learned in the past will allow for synthesis of new hypotheses toward our common goal of solving the riddle of Alzheimer's disease. Such efforts are imperative for the millions who suffer, for their families, and for the social and economic health of our society. As pointed out dramatically by Andrew Grove in a speech at the meeting of the Society for Neuroscience this year, a new and more productive approach to researching, discovering, and bringing to the public the news of effective treatments for neurodegenerative diseases is long overdue. This case report is published in an effort to broaden the spectrum of such potential treatment strategies for Alzheimer's disease, and for other diseases mediated by cytokines. Hopefully, our efforts will be supported by rapid allocation of the resources necessary for both basic and clinical scientific investigations that will answer Mr. Grove's revolutionary challenge.

## Competing interests

The author(s) declare that they have no competing interests.
